# Homicide among Indigenous females in North Carolina: a comparison of publicly generated data and violent death reporting system

**DOI:** 10.1093/fsr/owad057

**Published:** 2023-12-30

**Authors:** Muhammad Hudhud, Scott Proescholdbell, Tammy Norwood, Crystal Cavalier-Keck, Ronny A Bell

**Affiliations:** Department of Health Behavior, University of North Carolina – Gillings School of Global Public Health, Chapel Hill, NC, USA; Injury and Violence Prevention Branch, North Carolina Department of Health and Human Services, Raleigh, NC, USA; Injury and Violence Prevention Branch, North Carolina Department of Health and Human Services, Raleigh, NC, USA; Missing and Murdered Indigenous Coalition of North Carolina, NC, USA; Division of Pharmaceutical Outcomes and Policy, University of North Carolina – Eshelman School of Pharmacy, Chapel Hill, NC, USA

**Keywords:** homicide, Indigenous populations, North Carolina, women, injury and violence, prevention

## Abstract

Like other minoritized populations, American Indian/Alaska Native (AI/AN) females experience disparate morbidity and mortality outcomes to that of the general US population. This study identified discrepancies in reporting of AI/AN female decedents between the North Carolina Violent Death Reporting System (NC-VDRS) and an online, user-generated database. Female AI/AN decedent data of all ages were collected from the NC-VDRS and compared against that of the publicly available North Carolina Missing and Murdered Indigenous Women (MMIW NC) database for the study period, 2004–2019. Twenty-four of the 72 cases matched between data systems (33.3%). Substantive differences between the NC-VDRS and the MMIW NC database were found. Future efforts should be directed towards supporting Indigenous communities with the comprehensive data the NC-VDRS can provide. This paper highlights statewide public health systems like the NC-VDRS supporting community efforts to understand, advocate for, and disseminate information on MMIW.

## Introduction

The term “Missing and Murdered Indigenous Women” (MMIW) and its variants have been coined to underscore the historical destruction of native people and land that have persisted until this day. In recent years, government proclamations of 5 May as MMIW Day, and the creation of the Missing & Murdered Unit in the Department of the Interior have been efforts to address this public health issue (North Carolina (NC) Office of the Governor, 2021 [1]; US House of Representatives, 2021 [2]; US Department of the Interior, 2021 [3]). Indeed, while public awareness of MMIW has steadily grown, peer-reviewed literature is lacking. Relative to violent death, American Indian/Alaska Native (AI/AN) females living on reservations are 10 times more likely to be murdered than the general population (Centers for Disease Control, CDC) [4]. In fact, ~84% of AI/AN females experience some form of interpersonal violence [5]. The objective of this formative work study is to compare MMIW decedent data between systems, and, in turn, identify new avenues of research and ways to support prevention efforts.

## Data and methods

The North Carolina Violent Death Reporting System (NC-VDRS) is an incident-based surveillance system that collects data on deaths that result from violence in NC. The NC-VDRS is part of the CDC’s National Violent Death Reporting System (NVDRS). The NC-VDRS sources data from vital records (i.e. death certificates), law enforcement reports, and medical examiner reports. Per CDC guidance for NVDRS, ICD-10 codes U01-U02, X85-Y09, Y87.1 are required, for a classification of homicide in NC-VDRS.

The Missing Murdered Indigenous Coalition of North Carolina (MMIW NC) is a non-profit organization focused on advocacy, outreach, and reporting for missing and murdered women and girls. MMIW NC conducts programming for AI communities in NC around sexual violence education, understanding MMIW, and community healing and justice (available from www.mmiwnc.com).

The MMIW NC additionally maintains a publicly available database posted on its website in which anyone can submit a report for a missing or murdered individual (including male and two-spirit). That is, despite the focus on women, they maintain a database which contains cases of missing and murdered persons and is not necessarily restricted to female decedents, although the vast majority are female. In the MMIW NC database, cases are verified by the MMIW NC team through contact with the individual’s family, friends, community members, and local law enforcement. MMIW NC staff update the database as new frequently as data are obtained. The database relies on police reports to make determinations of homicide (i.e. murder). We compared female decedent data of all ages between the two datasets, matching variables that included name, incident year, death data, residence county, injury county, age, and race.

## Results

**Figure 1:**
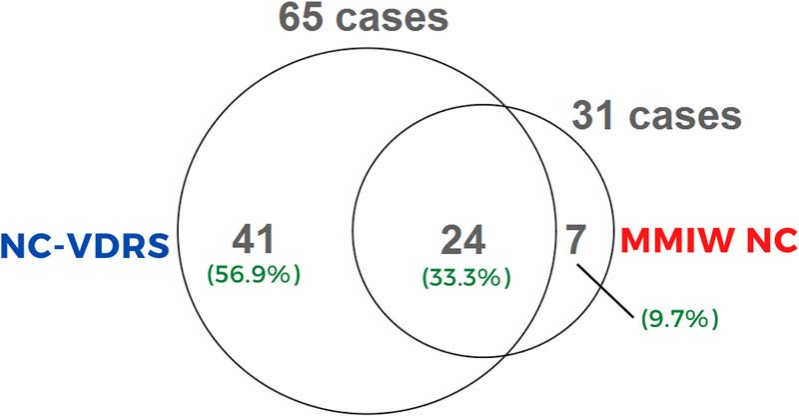
A visualization of the cases identified in North Carolina Violent Death Reporting System (NC-VDRS) and the North Carolina Missing and Murdered Indigenous Women (MMIW NC) database.

Sixty-five and 31 AI/AN female homicide decedents were identified in NC-VDRS and the MMIW NC databases, respectively (Figure 1). Twenty-four cases matched between datasets (33.3%). Seven cases available in MMIW NC database were identified in the NC-VDRS but not categorized as homicides (i.e. suicide, overdose, etc.); 41 cases in NC-VDRS were not available in MMIW NC’s public-facing database. The seven cases not categorized as homicides in the NC-VDRS were instead marked “undetermined” as the case definition of homicide was not met per the system’s classification criteria. One case of the seven was listed as White race and not AI/AN. MMIW NC staff were consulted regarding this case, and reported that the decedent, despite being White, was considered “culturally American Indian” due to their association with the AI community. Lastly, 12 of the 24 matched homicides occurred in the areas in which the Lumbee Tribe of NC is most populous (i.e. 10 in Robeson County, 1 in Hoke County, and 1 in Cumberland County).

## Discussion and conclusion

Our findings demonstrate substantive differences between a statewide surveillance system and that of a community advocacy organization. There are a few possible reasons for discrepancies between the datasets. First, some demographic data of AI/AN female decedents differed between datasets. Specifically, the MMIW NC dataset includes some non-AI decedents that were associated with, and culturally accepted as part of the AI community; as such, these individuals were classified as MMIW. In NC-VDRS, these individuals were listed as non-AI. In the MMIW NC dataset, race is not specifically defined. Rather, any decedent in the database is presumed to be of, or culturally associated with AI heritage. These data are verified through contact with the decedent’s family, friends, and community members. In NC-VDRS, race data are sourced from state vital records data.

Additionally, the case definition of homicide differs between datasets. The MMIW NC’s data are initially generated by an individual familiar with the decedent, with a team that tries to verify cases through contact with the decedent’s family, friends, community members, and law enforcement. The seven non-matched entries listed the manner of death as not a homicide in NC-VDRS. Instead, these cases were either “undetermined” or “unintentional poisoning” according to the final death certificate and medical examiner investigation.

It is worth noting that most homicides involving AI/AN individuals in the NC-VDRS and the MMIW NC databases occured among the areas where the Lumbee Tribe of NC is most populous, mainly to the east of the Mississippi River.

This study has a few limitations. First, the MMIW NC data are self-report. As such, the MMIW NC’s method of triangulating data to determine a homicide is reliant on whoever will speak to the team, and there is no single way to verify the accuracy of the information provided. Second, errors may have been introduced through the manual data extraction from the MMIW NC database, as there are no centralized, downloadable/exportable dataset on their website. Third, while the NC-VDRS did indeed include the seven cases not listed as a homicide in its master file, the “undetermined” status of each death is drawn from the source material by which the system is based upon. More specifically, because the law enforcement and medical examiner reports for these cases did not meet the criteria for deaths by violence, they have an “undetermined” cause of death. For these undetermined cases, death by violence is not completely ruled out, but they cannot be ascertained with the available information.

This work underscores the importance of systems like the NC-VDRS in its ability to synthesize its data sources for victims of homicide. The implications of this study are 2-fold. First, this study reinforces the need to investigate and address MMIW as a serious public health problem. Second, this study highlights how statewide surveillance systems like the NC-VDRS can better support Indigenous communities in quantifying and understanding MMIW and inform future prevention efforts. This preliminary work compared existing datasets capturing similar data.
